# Understanding the interests of academics from diverse disciplines to identify the prospective focus for a UK-based transdisciplinary network involving farm-to-fork stakeholders on antimicrobial resistance in agrifood systems: An online survey

**DOI:** 10.1016/j.onehlt.2024.100884

**Published:** 2024-08-26

**Authors:** K. Marie McIntyre, Maha Khan, Martha Betson, Lucy Brunton, Hernan Botero Degiovanni, Andrew P. Desbois, Mahmoud Eltholth, Paul Hurley, Lisa Morgans, John E. Pearl, Ruben Sakrabani, Orla Shortall, Katharina Watson, Jennifer Cole

**Affiliations:** a Modelling, Evidence and Policy Group, School of Natural and Environmental Sciences, Newcastle University, UK; b Department of Health Studies, Royal Holloway University of London, UK; c Department of Comparative Biomedical Sciences, School of Veterinary Medicine, University of Surrey, Guildford, UK; d Veterinary Epidemiology, Economics and Public Health Group, Department of Pathobiology and Population Sciences, Royal Veterinary College, University of London, UK; e Behavioural Change Group, REES, Scotland's Rural College (SRUC), Geosciences, University of Edinburgh, UK; f Institute of Aquaculture, Faculty of Natural Sciences, University of Stirling, UK; g Hygiene and Preventive Medicine Department, Faculty of Veterinary Medicine, Kafrelsheikh University, Kafr el-sheikh, Egypt; h School of Geography and Environmental Science, University of Southampton, UK; i Department of Agricultural Science and Practice, Royal Agricultural University, Cirencester, UK; j Department of Respiratory Sciences, University of Leicester, Leicester, UK; k School of Water, Energy & Environment, Cranfield University, Cranfield, UK; l Social, Economic and Geographical Sciences Department, James Hutton Institute, Aberdeen, UK

**Keywords:** Antimicrobial resistance, Agrifood, AMR evolution, AMR dissemination, Complexity, Farming, Mixed methods, Systems approaches

## Abstract

Antimicrobial resistance (AMR) evolution and onward transmission of resistance genes is impacted by interrelated biological and social drivers, with evidence and impacts observed across human, animal and environmental One Health domains. Systems-based research examining how food production impacts on AMR in complex agrifood systems is lacking, with little written on management approaches in the UK that might prevent and respond to this challenge. One approach is the creation of a transdisciplinary network to enhance capacity, capability and collaboration between agrifood-focused disciplines and stakeholders. This co-creation platform for network-wide systems-based activities would reduce inefficiencies in AMR-related activities around agrifood, providing a cross-cutting, cohesive community to deliver transformational guidance on relevant, practical agrifood solutions that add value by reducing AMR, antimicrobial usage and associated costs, and decreasing resultant environmental contamination by prioritising challenges, sharing knowledge and best practice, and co-creating practical solutions with key stakeholders. An online survey determined prospective network focus, structure and priorities, with responses analysed using mixed methods.

Survey results suggested respondents have interests in synthesising data using systems-approaches and using certain disciplines such as ‘social sciences’ within network activities. There were disconnects in how and whom to work with on this, with generalised use of ‘social science/scientists’ but lack of disciplinary understanding (e.g., anthropology, sociology) suggesting disciplinary differences awareness-training is useful. A similar generalisation is seen for mathematics/statistics. There are strong interests in working with food system practitioners (e.g., farmers/vets), providing opportunities for farm/field visits/knowledge exchange, and human health, reflecting the need for farm-to-fork understanding of impacts. There were notable mentions of policy/governance, emphasising translational research desires to create meaningful change. Disciplines/fields did not always align with identified interests e.g., systems and implementation science, suggesting the utility of network activity around introducing these disciplines e.g., methodology-focused rather than subject-focused conferences exploring lateral thinking about subjects. We suggest starting by developing understanding of the most important research questions by working with stakeholders, then working back to how we would achieve desirable project outcomes and who else is needed for this.

## Highlights

• Systems-based research lacks, examining food production impacts on AMR in UK agrifood systems.
• Solutions will add value; reducing AMR, AMU, associated costs, and environmental contamination.
• An online survey determined a transdisciplinary network's focus, structure and priorities.
• Disciplinary differences' awareness-training is vital – anthropology, sociology, maths, stats.
• Food system practitioner & policy/governance interest echo farm-to-fork translational impact.

## 1 Introduction

The evolution of antimicrobial resistance (AMR) and onward transmission of resistance genes is a ‘super wicked problem’ (one that is complex and time-dependent [1]) not limited to clinical environments. Interrelated biological and social drivers combined influence AMR [2], and evidence and impacts are seen across human, animal and environmental health domains. Research that examines food production roles on the evolution and dissemination of AMR is relatively lacking. It is thought, however, that most microorganisms, including AMR bacteria and genes, enter animals via water or soil [3], with their movement and spread in food production exacerbated by management practices such as irrigation with contaminated water supplies and animal manure used to fertilise land, which is then grazed by livestock or upon which crops, such as grains, vegetables and fruits, are grown. Onward transmission of bacteria then occurs from both crops and animals during food harvesting, preparation, handling and consumption, or through contact with animal faeces (i.e., from livestock and pets, e.g., from raw pet food) [2].

Since the United Nations Environment Programme (UNEP) joined the World Health Organization (WHO) [4], the Food and Agriculture Organization of the United Nations (FAO) [5] and World Organization for Animal Health (WOAH) [6] [7] in a quadripartite group addressing AMR, and following debate on the drivers and pathways of AMR evolution and onward transmission, UNEP has examined the role of the environment including agricultural and food production sectors in AMR ‘superbug’ development [8]. The most recent United Kingdom (UK) National Action Plan on AMR reviewed animal, plant and environment commitments including a target to reduce UK antibiotic usage in food-producing animals by 25 % between 2016 and 20; this was met ahead of schedule in 2018. Other proposed changes included optimising antimicrobial use (AMU) and improving data availability to better understand AMR prevalence across human and animal health domains, reducing need for and exposure to antimicrobials in all domains, minimising environmental spread and improving food safety around AMR [9]. Regulation and policy-making around AMR in the UK agrifood sector is covered by government agencies such as the Animal and Plant Health Agency [10], Food Standards Agency [11], Food Standards Scotland [12], Veterinary Medicines Directorate [13], and Department for Environment, Food and Rural Affairs [14], with help from organisations such as Responsible Use of Medicines in Agriculture Alliance [15], and Royal College of Veterinary Surgeons Knowledge AMR Hub [16]. Recent advances in understanding on AMR surveillance initiatives have been made by the Pathogen Surveillance in Agriculture, Food and Environment (PATH-SAFE) programme [17]. The UK also has an ability to act on international food producers at reach, as a high proportion of food products are imported to the UK (46 % of consumed products in 2020) [18] and thus has influence across international borders and along international supply chains. As a result, and given food production system complexities, there is an urgent need to prioritise areas of research that address management approaches to prevent and respond to AMR challenges in UK agrifood systems. An appropriate approach is the creation of a transdisciplinary network to enhance the capacity and capability of the UK research community by increasing collaboration between agrifood-focused disciplines and stakeholders, including practitioners, policy and industry groups. Such a community would provide a platform for co-created network-wide activities to improve understanding of this critical problem by ensuring that key stakeholder perspectives are fully integrated in problem-framing. This will supplement national and international AMR understanding, preparing us for future AMR challenges in UK agrifood systems by building new collaborations and proposing actions that enhance effective planning and mitigation.

The idea for such a network emerged in response to a funding call from the UK's Biological and Biomedical Sciences Research Council (BBSRC) to enable the creation of transdisciplinary networks to tackle the challenge of AMR [19]. To help determine a possible structure and priorities of such a network, an online survey was delivered to a multidisciplinary group of academics, commercial, governance and environmental stakeholders who had joined a consortium proposing a transdisciplinary network to enhance collaboration between agrifood stakeholder communities and research disciplines, and deliver network-wide activities that improve understanding of AMR challenges in UK agrifood systems. The survey aimed to explore the proposed focus of the network (limited by the conditions of the call to UK-based members), including consideration of food safety and food security, and aligned areas. We wanted to understand the knowledge and skills of respondents, and their ideas around the proposed network's agrifood foci, explore their expertise (research topics or disciplines), organisational focus, and the human, animal, plant and environment impacts examined in their work.

## 2 Methodology

Having developed (or been involved in developing) Expressions of Interest for prototype AMR networks, met at in-person AMR community meetings in summer 2023, and/or taken part in online discussions following these meetings, a group of 34 participants interested in AMR in agrifood systems was convened. In July 2023, an online questionnaire administered using JISC Online Surveys [20] (Table 1) was delivered to collect information on the group's interests; 30 participants responded. In Question 1, a closed question in which respondents selected from an options list, they provided the topics or disciplines their work covered and their human/animal/plant/environment foci*.* A second question, Question 1a, asked about other disciplines/topics of interest*.* Then, in three free-text fields (Questions 2, 2a and 2b) they were asked about their AMR interests and any specific research questions for interdisciplinary approaches, other disciplines they would work with on these interests/questions, and non-academic partners and networks needed to create a network of networks to maximise impacts of future network activities. Interdisciplinary rather than transdisciplinary approaches were explored as a first step in understanding of needs. Finally, in Question 3, potential network activities were ranked from most to least important, based on a £650,000 network budget over a 4-year period*.*

**Table 1:** Text used within survey questions.

Number	Question text
1	Select the topics or disciplines which your research/work examines and whether this is focused on impacts in humans, animals, plants and/or the environment? (please tick all that apply)
1a	Would you like to add any other disciplines/topics or further details to this list?
2	What are your interests in terms of AMR - do you have any specific research questions you would like to explore using interdisciplinary research and if so, what are they?
2a	Which other disciplines do you think you would work with to explore these interests/research questions?
2b	Are there any non-academic partners or networks you think you would work with to explore these interests/ research questions?
3	What sort of activities do you think an AMR network should develop – please rank from the most important to the least important

We analysed survey responses, collating the results from options provided for Questions 1, 1a and 3 (KMM), and using thematic coding for free-text fields in Question 2, 2a and 2b (JC). For Question 2a, respondents were asked to list disciplines the proposed network might work with, but their replies only sometimes referred to disciplines (e.g., ‘bacteriology’, line 3, column B; ‘economics’, line 27), and instead were as likely to describe an aim that implied a discipline (e.g. ‘understanding human and animal behaviour’, line 24, implying behavioural science), or broader fields or their practitioners (e.g. ‘environmental specialists’, line 9), or a methodology (e.g., ‘modelling’, line 5; ‘participatory approaches’, line 29). To address this, responses were coded to the UK research council considered most likely to fund research matching that description; AHRC (Arts and Humanities Research Council), BBSRC (Biological and Biomedical Sciences Research Council), ESPRC (Engineering and Physical Sciences Research Council), ESRC (Economic and Social Research Council), Innovate UK (Innovation research), MRC (Medical Research Council), NERC (Natural Environment Research Council), or STFC (Science and Technology Facilities Council). As assigning a response to a research council was to some extent subjective, this question was double-coded (by JC and MK, with KMM acting as arbiter in cases of disagreement). We acknowledge that this is a UK-centric approach and that UK research councils may have no direct equivalent in other countries or regions, but as only UK-based researchers were eligible to join the network, we feel that this is a reasonable categorisation to follow. Further information on the remits of the Councils funded by UK Research and Innovation (UKRI) is available [21].

## 3 Results

**Fig. 1:**
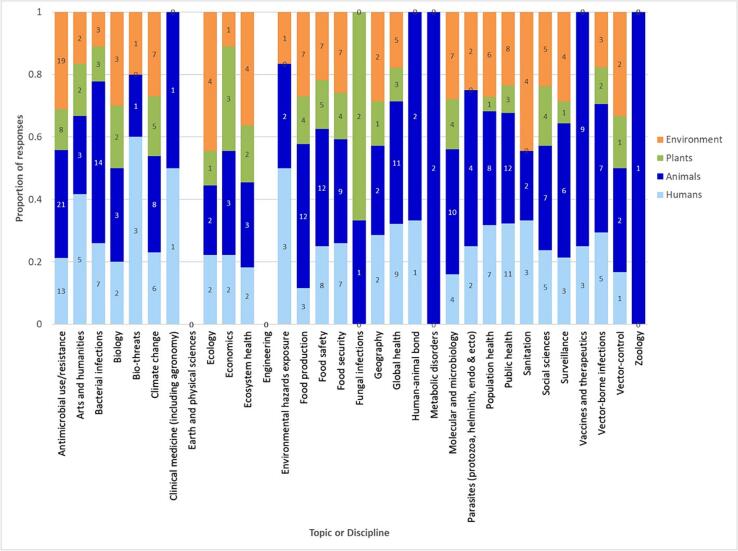
Topics or disciplines which survey respondents work on and whether they are focused on humans, animals, plants and the environment.

Responses to Question 1 recorded experience of most topics and disciplines within the group, with a spread of examining impacts in humans, animals, plants and the environment (Fig. 1). It is recognised that not all human, animal, plant and environment impact groups are appropriate for every topic or discipline (e.g., for plants, human-animal bond, metabolic disorders, sanitation and zoology are probably not relevant). Plants were least often the focus of research, being particularly poorly or not represented for bio-threats, agronomy, environmental hazards exposure, parasites (including protozoa, helminths, endoparasites and ectoparasites), and sanitation. Additional topics and disciplines (Question 1a) were suggested including some focused on interdisciplinary research approaches and translational understanding, e.g., action research, education and stewardship, ethics, mitigation strategies, One Health and policy (Table 2).

**Table 2:** Frequency of topics or disciplines which survey respondents work on in addition to those specified in the survey.

Other topics/disciplines	Frequency
Action Research approaches	1
Agriculture	3
AMR detection	1
Bacteriophage in biocontrol (and general phage-virome)	1
Education/Stewardship	1
Ethics	1
Genomics/Population genetics	2
Livestock systems	1
Mathematical modelling	2
Mitigation strategies	1
One Health	1
Policy	1
Selective pressures/Evolution (in the environment)	2
Soil science	2

Question 2 was coded into three thematic categories that emerged from the responses (‘subjects’, ‘approaches’ and ‘impacts’). The category ‘subjects’ was then divided into four sub-categories in the second coding round: drivers, reservoirs, movement and novel entities. Respondents felt that examining the evolution and ecology of AMR in agrifoods was particularly important, including identifying the reservoirs and drivers of risk; co-selective effects of AMU, and risks arising from evolution, movement and spread of AMR in food animals, bacteria, AMR genes, and mobile genetic elements. Novel entities such as alternative foods and crops were also highlighted, in addition to emerging technologies. Overall, the theme for ‘subjects’ can be described as ‘evolution and ecology’.

The ‘approaches’ category was also divided into four sub-categories: systems-approaches, behaviour change approaches, policy approaches and economic approaches. These suggested interests in synthesising data using systems (including One Health or Planetary Health frameworks) and in using social science methods, undertaking food-system mapping and monitoring exercises. Change management and behaviour change approaches were important to address knowledge gaps, identify barriers to change and their cultural contexts. Interaction with policy-makers and stakeholders across the food supply chain was seen as vital to identify research questions, with outputs being policy-facing and tailored to regulation and control of AMR. Finally, economic consideration in intervention planning was important to incentivise interventions and understand disincentives to change; again, supported by change management approaches.

The ‘impacts' category did not have any emergent sub-categories, but respondents were interested in selective pressures on AMR, the impacts of climate change, and how power imbalances between food system stakeholders have effects across agrifood systems. This suggests an important role for implementation scientists and stakeholders, who have experience of developing interventions and understanding their impacts, within the proposed AMR agrifood network, and in turn reflects respondents' suggestions for additional topics and disciplines.

Question 2a, which examined other disciplines the proposed network might work with, proved more challenging to code, as discussed above. To make sense of this, free-text responses were extracted from the table and each reference to a discipline, methodology, field or practitioner was listed in alphabetical order, with the number of mentions recorded. In total, 121 discrete references were extracted, resulting in 85 different first-level category codes (as some references occurred more than once, e.g., ‘plant science’ was mentioned by three separate respondents, and ‘economics’ was mentioned by six). The total number of category codes was greater than the number of respondents as many respondents made more than one reference (e.g., line 29, makes three: “social sciences, mathematical modelling, participatory approaches”). For the second-stage coding, the responses were categorized by the UK research council to which each was best matched (e.g., ‘participatory approaches’ and ‘anthropology’ were both coded to AHRC; ‘human health specialists’ and ‘clinical infectious disease’ to MRC). As such coding is subjective and vulnerable to bias, this second-stage coding was undertaken independently by two coders (JC and MK) and differences in category code assigned were resolved by a third (KMM). Resolution could itself be difficult, as more than one research council may legitimately provide funding (e.g., antimicrobial development could be funded by MRC or Innovate UK). In such cases, the reference was double-counted to both councils. Some topics mentioned were considered completely cross-council, including ‘AMR risk’, ‘climate science’ and ‘net zero’; these were not coded to a research council but are noted for future reference. This process resulted in 125 items, coded against the 85 first-level codes, and then coded to a research council category in the second coding round. This categorized the responses as relating to topics funded by BBSRC (*N* = 34), ESRC (*N* = 27), NERC (*N* = 20), MRC (*N* = 19), EPSRC (*N* = 12), AHRC (*N* = 10), Innovate UK (N = 2) and STFC (N = 1). Where discrete disciplines to engage with were named, the most popular were social scientists (N = 10), economists (*N* = 9), environmental scientists (*N* = 6) and veterinarians (N = 6), but with representation of many others, including modellers, policy scientists, molecular biologists, food supply chain analysts and engineers. Respondents wanted to work across agriculture/plants, climate change and the environment, human health, economics, policy and governance, and many other fields, with many describing skills-sets or approaches, e.g. data extraction, impact, prediction, risk, supply chains and systems thinking, not all of which were possible to code to a single research council, supporting the need for cross-council approaches to AMR.

Question 2b (*“Are there any non-academic partners or networks you think you would work with to explore these interests/ research questions?*”) was coded by first extracting text from the free-text field, listing all organisations mentioned in alphabetical order and then thematically coding them against the emergent categories ‘farmers/farming unions’ (*N* = 15), ‘clinical veterinary organisations’ (*N* = 9), ‘food standards agencies/organisation’ (*N* = 8) and ‘fisheries/aquaculture organisations’ (including Defra, the UK Government Department for the Environment, Food and Rural Affairs, N = 8), ‘animal health/veterinary training/membership/livestock organisations’ (*N* = 7), ‘environment organisations and agencies’ (*N* = 6), ‘human health agencies (N=4)’, ‘agriculture agencies/institutes’ (*N* = 3), ‘AMR/antimicrobial usage (AMU) surveillance programmes’ (N = 3), ‘stakeholders providing data’ (*N* = 2), and ‘AMR/AMU stewardship programmes’ (*N* = 1). The ‘Others’ category included agribiotech companies, artists, the water industry and waste management companies. Survey respondents were interested in working with a broad mixture of non-academic partners and networks largely focused on practitioners (including mentioning farmers and vets) and government agencies; food standards and environment agencies were also prominent.

**Fig. 2:**
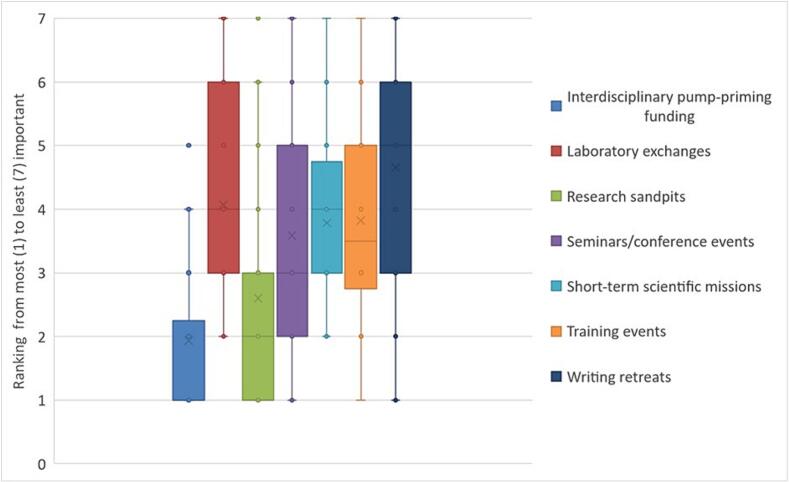
Activities respondents felt the network should develop ranked by importance from most [1] to least [7].

In Question 3: The most important network activities ranked by respondents, based on the median value provided within the ranking and the range of values around the median, were delivering transdisciplinary pump-priming funding schemes, followed by research sandpits; seminars and conferences were seen as important but there was disparity in their importance (a large range in where they were ranked), and their overall ranking was closely aligned to that of short-term scientific missions and training events. Finally, the greatest spread of opinion was on the importance of laboratory exchanges and writing retreats; the latter had a higher median scoring, suggesting that the respondents see it as less important than the other potential activities. (Fig. 2).

## 4 Discussion

Our analyses describe a template upon which we can build, for a proposed network to provide an overview of the focus of AMU and AMR across the animal, environmental and human domains in agrifood systems. The network will identify the main questions, knowledge gaps and networks' strengths and weaknesses. It will provide strategic, cohesive direction, harmonising the research activities of academics focused on increasing understanding of AMR in agri-foods and acting as an integrator and ‘force-multiplier’ with stakeholders, building on the approach and findings of other networks and from previous analyses e.g., focused on Asia [22]. The resulting co-creation platform for network-wide systems-based activities will add value to current siloed approaches, reducing inefficiencies in AMR-related activities around agrifood, providing a cross-cutting, cohesive community to deliver transformational guidance on relevant, practical agrifood solutions by reducing AMU, AMR and associated costs, and decreasing resultant environmental contamination. The next analysis phase will include extending the population sampled, seeing how well various sectors are currently represented in the network and where obvious gaps lie. This directly led to an initial network objective to undertake a mapping exercise to see how closely membership matches to what and who respondents in an initial network want to work with; this will be delivered at the first formal network meeting, which we estimate will take place before the end of 2024. The mapping exercise will be supplemented by examining the activities of aligned networks e.g., the Microbes and Social Equity Working Group, which aims to connect microbiology with social equity research, education, policy, and practice [23]; the AMR Knowledge Hub [24], which provides resources and information to support researchers and practitioners globally; The Joint Programming Initiative on Antimicrobial Resistance Virtual Research Institute (JPIAMR-VRI), which aims to increase or improve AMR research coordination, visibility of AMR research networks, research performing institutes/centres and infrastructures, and global knowledge exchange and capacity development [25] and other JPIAMR-funded networks, see [26] and projects e.g., [27,28].

It was noticeable in results that while specific scientific disciplines were mentioned, (e.g., ‘molecular biology’, ‘bacteriology’), most social science discipline mentions were more general (e.g., just ‘social science’) with far fewer specific disciplines described, e.g., anthropology, sociology. Behavioural science methodology was mentioned but not ‘behavioural science’ as a field or discipline (e.g., Line 31 asks “How can we best influence behaviours in farming and wider society?” but then lists ‘Policy, food supply chain science/economics/management, AgriTech and engineering’ as disciplines). This use of the generalised terms ‘social science/scientists’ suggests that while network members showed a clear desire to work with social scientists, some awareness-training in the differences between social science disciplines and what each can offer would be valuable, especially in light of a recent scoping review that suggests that only around a quarter of papers discussing AMU in veterinary and animal science journals incorporate social science approaches and methodologies to understand reasons for that use [29]. This might take the form of exploratory workshops and seminars, perhaps with case study examples from previous or existing projects of social science's inputs and impacts. The discussion of ‘social science/scientists’ also reflects the idea that social science domains are generally more self-contained, making it difficult for knowledge from other disciplines to flow into them; this compares to scientific disciplines from which knowledge can be more easily accessed [30]. The methodologies mentioned include behaviour and engagement (*N* = 5), data (N = 5), epistemology (*N* = 3) and laboratory techniques (*N* = 1). This suggests that several respondents wanted to ‘*understand [x]*’ but were using vague terms to describe outcomes rather than naming specific methodologies, such as Participatory Rural Appraisal [31] or Actor Network Theory [32]. Network activities could help disciplines to understand one another better. A similar generalisation was seen for mathematics/statistics, where people wanted to ‘do things with data’ but didn't specify what, or with who. This compared to disciplines from which researchers interested in AMR traditionally come; biological and biomedical disciplines and fields such as epidemiology, bioinformatics, bacteriology, which were more likely to be referred to in specific terms.

The spread of fields mentioned by respondents suggests desires to work both with people who understand plants, the environments in which plants grow, and food systems or chains. It might also infer opportunity for running farm visits and field trips, additional to knowledge exchange activities within the proposed network. In terms of clinical sciences, there was a large interest in human health, reflecting the need to join up understanding across the food system, including into hospital and public health environments, with less focus on laboratory processes. There were also notable mentions of policy or governance, emphasising the need and desire for translational research, and providing a route towards meaningful change.

It was interesting that disciplines and fields did not align closely necessarily with the interests identified, i.e., while there was clear interest in systems science, implementation science in ‘Interests’ (Q3), respondents did not automatically list disciplines which are likely to have people with these skills as ones they wanted to work with (in Q3a). This might lend itself to some network activity around introducing these disciplines (and people who work in them) and discussing what can and cannot be done, and how this might be useful, e.g., food systems scientists describing how they apply systems theory to food chains; and anthropologists describing Actor Network Theory to determine the most influential roles in networks/systems. Systems engineers would be useful to describe how to configure systems for optimal throughput and most efficient output.

In terms of non-academic partners or networks that survey respondents wanted to work with, there was no explicit mention of organisations associated with change management, despite several suggestions implying a need to understand and use this method.

In this study, we have observed interests in synthesising data using systems-approaches and using certain disciplines such as ‘social scientists’ within proposed network activities. There are, however, disconnects in understanding on how and whom to work with to achieve this. There is also strong interest across the respondents in working with food system practitioners (e.g., farmers, vets) and government agencies in co-produced research to ensure that academics fully understand the problems practitioners face and that solutions proposed by academics are practical and implementable in practice. One network activity could be methodology-focused rather than subject-focused conferences in which different ways of thinking about subjects can be explored. We will start by developing understanding of the most important research questions by working with such stakeholders, and then working back to how we will achieve desirable project outcomes and who else is needed for this. Following the work undertaken, an AMR in Agrifood Systems Transdisciplinary (AMAST) network was proposed (Table 3) and a bid to fund such a network submitted, successfully, to the BBSRC. AMAST will officially begin activity in summer 2024 and we welcome approaches by researchers who would like to engage; we expect AMAST to provide a platform for future non-UK expansion.

**Table 3:** Main aims of the AMR in Agrifood Systems Transdisciplinary (AMAST) network.

A1.	To *create* a transdisciplinary network that enhances collaboration between agrifood stakeholder communities and diverse academic research disciplines to deliver network-wide activities that improve the understanding of AMR challenges in UK agrifood systems. Amongst the UK research community, AMAST will take a broad view of research disciplines that can collectively increase the capacity and capability to generate and answer priority questions on AMR in UK agrifood systems, including across production systems such as crop, livestock and aquaculture production and their contextual environments, and considering impacts from production through to impacts in consumers.
A2.	To *harness* key stakeholder perspectives into the problem-framing of AMR agrifood threats and opportunities during AMAST engagement exercises designed to capture, co-produce and share new knowledge within the network. This collective understanding will allow the AMAST collaboration to identify and prioritise areas of opportunity and areas of need for research that can inform new pathways to positively impact AMR.
A3.	To *prepare* for prioritised AMR challenges in UK agrifood systems by increasing understanding of these systems, developing informative metrics to quantitatively describe components of these systems, strengthening partnerships, and developing opportunities and a critical mass to solve AMR problems within new transdisciplinary teams, particularly those involving ECRs and disciplines that may not previously have engaged with AMR as a challenge. The AMAST collaboration will propose new systems-level transdisciplinary research and partnership frameworks and will use communication strategies to share these and other creative multimedia outputs broadly and publicly to support awareness and capacity development.

## Authorship

K.M. McIntyre, M. Betson, P. Hurley, L. Morgans and J. Cole made substantial contribution to the conception and design of this work. K.M. McIntyre and J. Cole wrote the manuscript. K.M. McIntyre, M. Khan and J. Cole made the substantial acquisition, organization, and interpretation of the data. All authors have read and approved the submitted version of the manuscript and have agreed to be personally accountable for the author's own contributions and to ensure that questions related to the accuracy or integrity of any part of the work, even those in which the author was not personally involved, were appropriately investigated and resolved, and the resolution documented in the literature.

## Funding statement

The authors thankfully acknowledge their individual funders for their support for this work, including the 10.13039/501100000354Food Standards Agency, Quadram researchers, the BBSRC, and the 10.13039/501100000691Academy of Medical Sciences (AMS). The findings and conclusions of this study are those of the authors and do not necessarily reflect the positions or policies of their funders. For open access, the authors have applied a CC BY public copyright license to any Author Accepted Manuscript version arising from this submission.

## CRediT authorship contribution statement

**K. Marie McIntyre:** Conceptualization, Data curation, Formal analysis, Methodology, Writing – original draft, Writing – review & editing. **Maha Khan:** Data curation, Formal analysis, Writing – review & editing. **Martha Betson:** Conceptualization, Methodology, Writing – review & editing. **Lucy Brunton:** Writing – review & editing. **Hernan Botero Degiovanni:** Writing – review & editing. **Andrew P. Desbois:** Writing – review & editing. **Mahmoud Eltholth:** Writing – review & editing. **Paul Hurley:** Conceptualization, Writing – review & editing, Methodology. **Lisa Morgans:** Conceptualization, Writing – review & editing, Methodology. **John E. Pearl:** Writing – review & editing. **Ruben Sakrabani:** Writing – review & editing. **Orla Shortall:** Writing – review & editing. **Katharina Watson:** Writing – review & editing. **Jennifer Cole:** Conceptualization, Data curation, Formal analysis, Methodology, Writing – original draft, Writing – review & editing.

## Declaration of competing interest

None of the authors have competing financial or non-financial interests in the writing of this manuscript.

## Data Availability

The data supporting the findings of this study are contained in the manuscript.
